# High serum haptoglobin level is associated with tumor progression and predicts poor prognosis in non-small cell lung cancer

**DOI:** 10.18632/oncotarget.9676

**Published:** 2016-05-27

**Authors:** Jianjun Lu, Yanhong Wang, Miansheng Yan, Pinning Feng, Linjing Yuan, Yuesu Cai, Xin Xia, Min Liu, Jinmei Luo, Laisheng Li

**Affiliations:** ^1^ Department of Laboratory Medicine, The First Affiliated Hospital of Sun Yat-Sen University, Guangzhou, 510080, People's Republic of China; ^2^ Department of Thoracic Surgery, The First Affiliated Hospital of Sun Yat-Sen University, Guangzhou, 510080, People's Republic of China; ^3^ Department of Internal Medicine, Medical Intensive Care Unit and Division of Respiratory Diseases, Third Affiliated Hospital of Sun Yat-Sen University, Guangzhou, 510630, People's Republic of China; ^4^ Department of Gynaecology, The First Affiliated Hospital of Sun Yat-Sen University, Guangzhou, 510080, People's Republic of China; ^5^ Institute of Laboratory Medicine, Guangdong Medical University, Dongguan, 523808, People's Republic of China

**Keywords:** haptoglobin, NSCLC, progression, prognosis, biomarker

## Abstract

The overall survival time of non-small cell lung cancer (NSCLC) has not improved dramatically in recent decades. An important reason is the lacking of valuable biomarkers. Haptoglobin was reported to have activities of anti-inflammatory, anti-oxidant, autoimmune and tumor angiogenesis. However its potential role as a tumor biomarker was not well recognized. We used an immunoturbidimetry method to measure serum haptoglobin levels in 205 NSCLC patients, and 210 normal healthy controls. We found that serum haptoglobin levels were significantly elevated in NSCLC patients compared with normal healthy controls (1.985±1.039 mg/mLvs. 0.922 ± 0.495 mg/mL, respectively, *P* < 0.0001). Higher serum haptoglobin levels were associated with advanced TNM stage, lymph node metastasis, and distant metastasis. Area under receiver operating characteristic curve (ROC) for serum haptoglobin was 0.809 (95% CI: 0.767–0.852) at a specificity of 0.881 and sensitivity of 0.639. The optimal cut-off value of haptoglobin was 1.495 mg/mL for discriminating NSCLC from normal healthy controls. Kaplan-Meier log rank analysis revealed that the higher serum haptoglobin levels group had a poorer overall survival compared with lower haptoglobin group (the median survival was 12.0 weeks, 26.0 weeks, respectively, *P* < 0.01). Further univariate and multivariate Cox regression analysis showed that serum haptoglobin was an independent risk factor of prognosis of NSCLC patients (*P* < 0.01, *P* = 0.01, respectively). In conclusion, our study suggests that serum haptoglobin may act as useful clinical serological biomarkers in progression and prognostic evaluation in NSCLC.

## INTRODUCTION

Lung cancer remains the most frequent incident cancer and the premier cause of cancer-related mortality for both men and women in China [1]. The most commonly diagnosed lung cancers are non-small cell lung cancer (NSCLC), which accounts for up to 85%. There are three histological subtypes of NSCLC, adenocarcinoma, squamous cell carcinoma, and large cell carcinoma [2]. NSCLC prognosis is deeply hinge on early stage diagnosis, more than 50% of patients with early stage diseases survive more than 5 years, compared with 5 year survival rate of less than 5% in patients with advanced stage diseases [3]. These lead many physicians and investigators to hunt for early screening strategies, including novel biomarkers and diagnostic tools to detect early stage NSCLC. However, those examination methods including imaging with chest X-rays, low dose computed tomography scans [4], sputum exfoliative cytological analysis, and a series of serum biomarker, such as CEA, NSE, CYFRA21-1, and SCC [5], have little to do with displaying the mortality of lung cancer. Therefore, investigation of a novel biomarker is clinically urgently needed.

Haptoglobin, an acute phase reactant protein, mainly produced by liver but also by other tissues, such as lung, spleen, kidney, skin and adipose tissue [6], which is constituted by an alpha chain and a beta chain, displaying in humans as three major phenotypes, Hp1-1, Hp2-2 and Hp2-1 [7]. It mainly functioned in the area of anti-inflammatory and anti-oxidant, but also in the field of autoimmune diseases, neurodegenerative diseases, and tumor angiogenesis [8, 9]. Elevated levels of serum haptoglobin were found in patients with inflammatory diseases [10] and a variety of cancers, including lung cancer [11–14]. We supposed that the serum levels of haptoglobin might be a good biomarker for progression and prognosis of NSCLC patients. However, to date, there is no report about the association between serum haptoglobin levels and prognosis of NSCLC.

In this study, to investigate the application of serum haptoglobin as a diagnostic potential biomarker for NSCLC, we measured the levels of serum haptoglobin in NSCLC patients by turbidimetric immunoassay and validated their ability to predict prognosis in NSCLC.

## RESULTS

### Clinical characteristics of patients

In this study, we recruited a total of 205 NSCLC patients and 210 normal healthy controls. The demographic, pathologic, and clinical information of the study subjects were displayed in Table 1. The mean age of the NSCLC patients (61.6, years) was not obviously different from normal healthy controls (60.6, years). The proportion of male gender accounted for 65.4% of the NSCLC patients and 57.1% of the normal healthy controls, respectively, with no significantly difference. However, the proportion of smoking status was apparently more in NSCLC patients than normal healthy controls, which was 71.7% vs. 56.2%. More than half of the patients were in stage III + IV (70.2%), 63.4% of the patients with lymph node metastases, and 24.4% with distant metastases.

**Table 1:** Clinicopathological variables of NSCLC patients and normal healthy controls (*n*, %)

Variables	NSCLC cases (*n* = 205)	Normal controls (*n* = 210)
Age (years)	61.6 ± 9.8	60.6 ± 11.1
Gender (*n*, %)		
Male	134 (65.4)	120 (57.1)
Female	71 (34.6)	90 (42.9)
Smoking status (*n*, %)		
Non-smoker	58 (28.3)	92 (43.8)
Smoker	147 (71.7)	118 (56.2)
Histology		
Adenocarcinoma	116 (56.6)	
Squamous cell carcinoma	76 (37.1)	
Other	13 (6.3)	
TNM stage		
I+II	61 (29.8)	
III+IV	144 (70.2)	
Differentiation		
Well	46 (22.4)	
Moderate and low	144 (70.3)	
Unknown	15 (7.3)	
Lymph node metastases		
Negative	75 (36.6)	
Positive	130 (63.4)	
Distant metastases		
Negative	155 (75.6)	
Positive	50 (24.4)	

### Association between serum haptoglobin levels and clinicopathological variables

**Figure 1:**
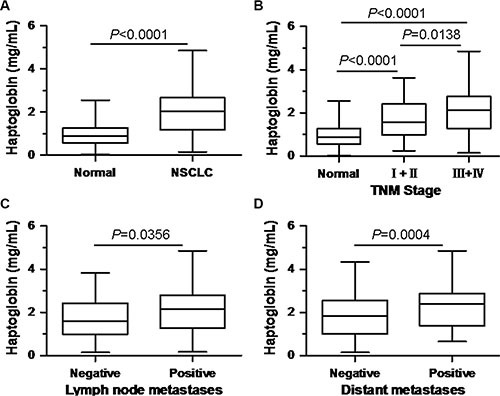
Comparison of serum haptoglobin levels (**A**) between normal healthy controls and NSCLC patients; (**B**) in normal healthy controls and NSCLC patients at different TNM stage; (**C**) in NSCLC patients with and without lymph node metastasis; (**D**) in NSCLC patients with and without distant metastasis.

To determine the potential of haptoglobin as a serological biomarker for NSCLC, we analyzed the levels of serum haptoglobin in NSCLC patients and normal healthy controls by turbidimetric immunoassay. The mean serum haptoglobin level in the NSCLC patients (1.985 ± 1.039 mg/mL) was significantly elevated, compared with the normal healthy control group (0.922 ± 0.495 mg/mL) (*P* < 0.0001, Figure 1A). The statistical power value was 1.0 (> 0.8 was considered significant). We further evaluated the clinicopathologic significance of the serum haptoglobin level in NSCLC patients. Table 2 summarized the association between the serum haptoglobin levels and clinicopathological variables in NSCLC patients. As displayed in Figure 1B, when compared with the normal healthy controls, the serum haptoglobin were notably elevated in NSCLC patients at both early TNM stage (stage I + II) and advanced TNM stage (stage III + IV), and serum haptoglobin level was even higher in advanced stage patients than early stage patients. Furthermore, the serum haptoglobin levels were obviously higher in patients with lymph node metastases than those without (2.136 ± 1.077 mg/mL, 1.719±0.917 mg/mL, respectively, *P* = 0.0356) (Table 2, Figure 1C). Meanwhile, statistically significant differences in haptoglobin levels were found between NSCLC patients with distant metastases and those patients without distant metastases (2.354 ± 1.069 mg/mL, 1.867 ± 1.005 mg/mL, respectively, *P* = 0.0004) (Table 2, Figure 1D). Besides, the serum haptoglobin levels were observed have no significant differences from other clinicopathological variables (Table 2). After all, these results indicated that serum haptoglobin levels increased in NSCLC patients, and associated with the progression and metastasis NSCLC, which could be serve as a potential biomarker to differentiate NSCLC patients from healthy controls, even the indicator for prognosis.

**Table 2:** Association between serum Haptoglobin levels and characteristical variables in NSCLC patients

Variables	Number	Haptoglobin (mg/mL)	*P* values
Age (Y)			
≤ 60	121	1.976 ± 1.098	0.9240
> 60	84	1.996 ± 0.972	
Gender			
Male	134	1.971 ± 1.029	0.6071
Female	71	2.014 ± 1.066	
Smoking status			
Non-smoker	58	1.889 ± 0.920	0.6772
Smoker	147	2.023 ± 1.084	
Histology			
Adenocarcinoma	116	2.009 ± 1.023	0.8547
Squamous cell carcinoma	76	1.964 ± 1.045	
TNM stage			
I + II	61	1.675 ± 0.881	0.0138
III + IV	144	2.117 ± 1.076	
Differentiation			
Well	46	1.964 ± 0.954	0.8732
Moderate and low	144	1.993 ± 1.052	
Lymph node metastases			
Negative	74	1.719 ± 0.917	0.0356
Positive	131	2.136 ± 1.077	
Distant metastases			
Negative	155	1.867 ± 1.005	0.0004
Positive	50	2.354 ± 1.069	

### ROC analysis of serum haptoglobin levels in NSCLC patients

**Figure 2:**
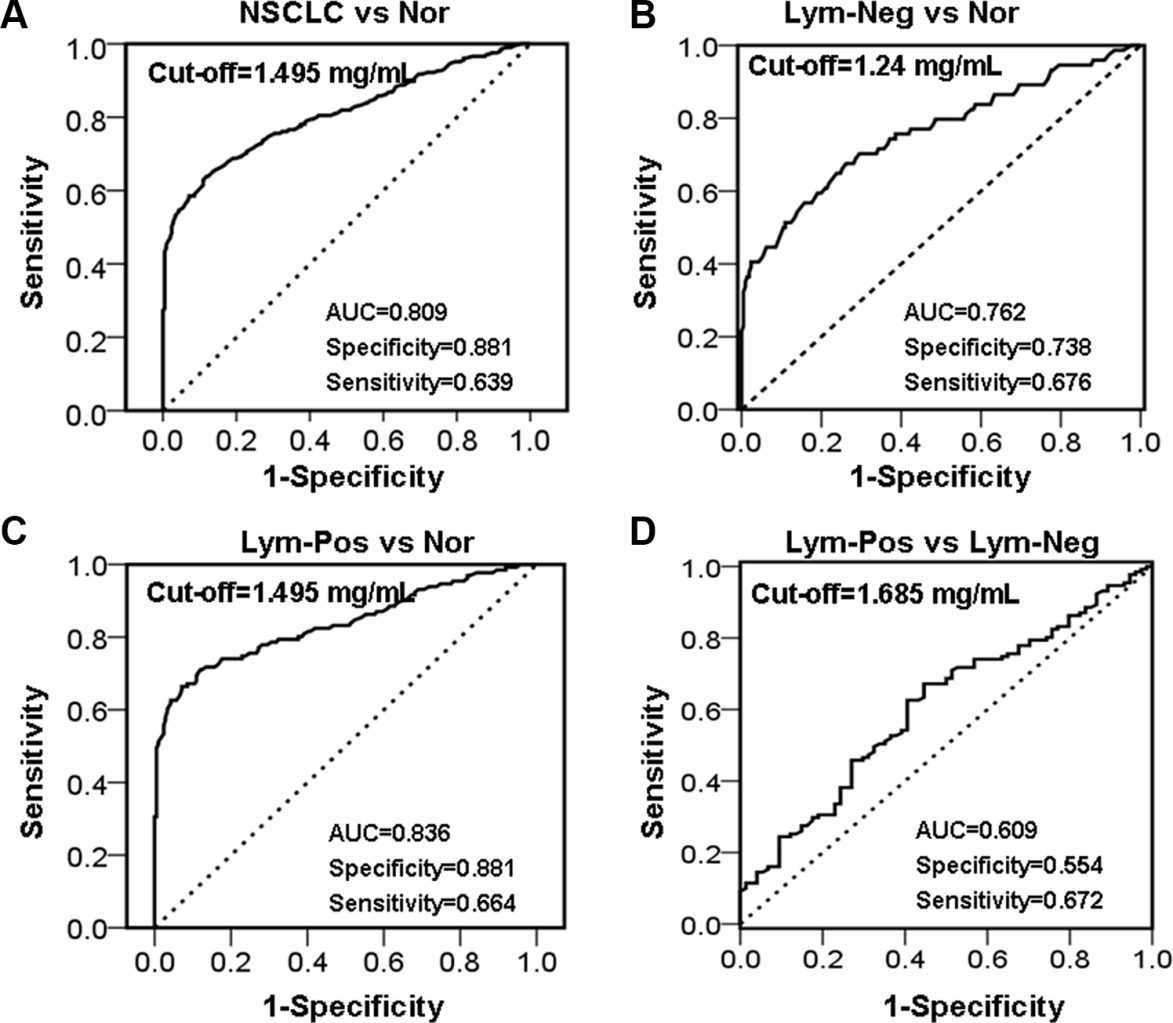
ROC analyses for serum haptoglobin to differentiate (**A**) NSCLC from normal healthy controls; (**B**) NSCLC patients with Lym-Neg from Nor; (**C**) NSCLC patients with Lym-Pos from Nor; (**D**) NSCLC patients with Lym-Pos from Lym-Neg. Nor, Normal healthy controls, Lym-Neg, Lymph node metastasis negative, Lym-Pos, Lymph node metastasis positive.

To evaluate the value of serum haptoglobin as a biomarker for NSCLC diagnosis, we calculated the ROC/AUC through plotting sensitivity against specificity at different sort for serum haptoglobin. At the beginning, we assess the value of serum haptoglobin for discriminating NSCLC patients from normal healthy controls, ROC/AUC analysis displayed a sensitivity of 0.639 (specificity of 0.881, AUC=0.809, 95% CI: 0.767–0.852, cut-off value = 1.495 mg/mL, Figure 2A). Then, in order to discriminate NSCLC patients without lymph node metastases from normal healthy controls, ROC/AUC analysis reached a sensitivity of 0.568 (specificity of 0.843, AUC = 0.762, 95% CI: 0.692–0.832, cut-off value = 1.405 mg/mL, Figure 2B). We further to distinguish NSCLC patients with lymph node metastases from normal healthy controls, ROC/AUC analysis showed a sensitivity of 0.664 (specificity of 0.881, AUC = 0.836, 95% CI: 0.788–0.884, cut-off value = 1.495 mg/mL, Figure 2C). Last, to predict NSCLC patients lymph node metastasis status, we compare the NSCLC patients with lymph node metastasis or not, data presented a sensitivity of 0.672 (specificity of 0.554, AUC = 0.609, 95% CI: 0.530–0.688, cut-off value = 1.685 mg/mL, Figure 2D). After all, we use serum haptoglobin levels at 1.495 mg/mL as cut-off value of NSCLC for subsequently analysis.

### Serum haptoglobin levels are an independent prognostic indicator for overall survival of NSCLC patients

**Figure 3:**
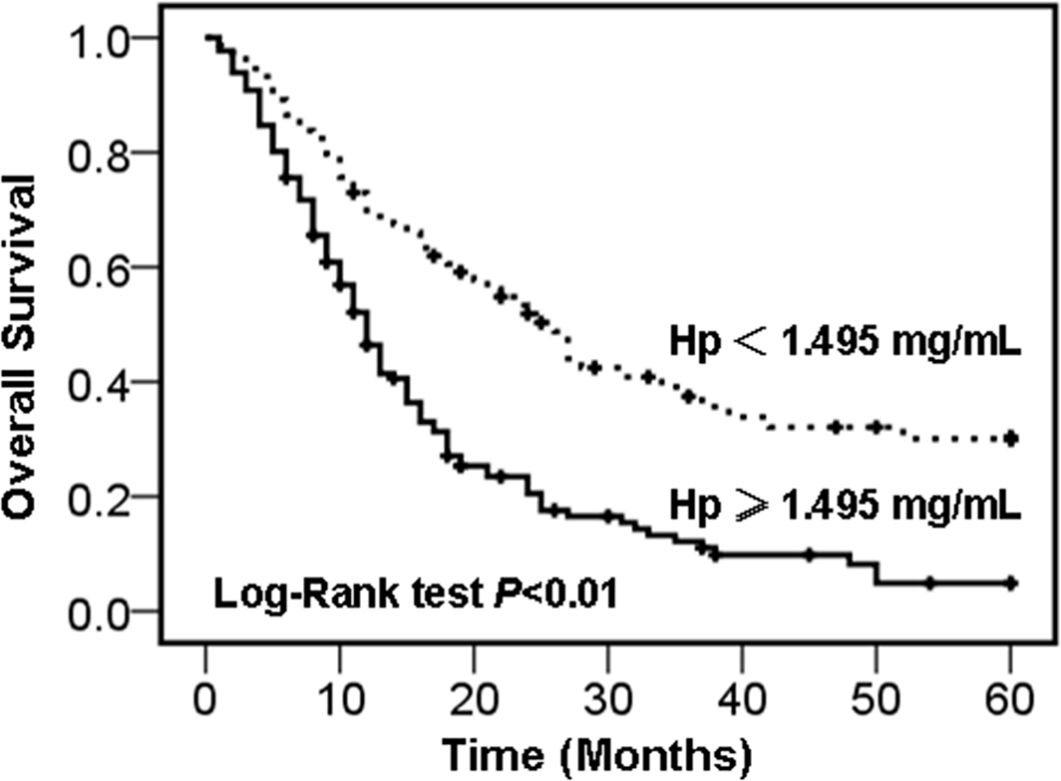
Kaplan-Meier survival curves of NSCLC patients Overall survival rate of NSCLC patients with high- and low- serum haptoglobin levels group.

To evaluate the prognostic significance of the serum haptoglobin levels, we used serum haptoglobin cut-off value 1.495 mg/mL, which was calculated from previous ROC analysis, as a threshold to partitioned 205 NSCLC patients into two groups, high serum haptoglobin group (haptoglobin ≥ 1.495 mg/mL, *n* = 131) and low serum haptoglobin group (haptoglobin < 1.495 mg/mL, *n* = 74). Overall survival time was calculated from the date of sampling to the date of death from cancer (cancer-specific survival). Deaths caused from not cancer-related, unknown factors, and subjects alive were censored. As shown by Kaplan-Meier log rank analysis, the higher serum haptoglobin levels group was correlated with a poorer overall survival, compared with lower serum haptoglobin levels group, the median survival time were 12.0 weeks (95% CI, 10.3 to 13.7) and 26.0 weeks (95% CI, 20.3 to 31.7), respectively, (*P* < 0.01, Figure 3). Further analysis using univariate and multivariate Cox regression showed that TNM stage, lymph node metastasis status, pathological distant metastasis status, and serum haptoglobin levels were independent risk factors of prognosis of NSCLC patients (Table 3).

**Table 3:** Univariate and multivariate Cox analysis of variables considered for overall survival rates of NSCLC patients

Variables	Category	Univariate *P* value	HR	Multivariate 95% CI	*P* value
Age	> 60 vs. < 60 years	0.45	1.07	0.52–2.18	0.37
Gender	male vs. female	0.63	0.95	0.58–1.68	0.26
Smoking status	smoker vs. non-smoker	0.32	1.18	0.34–2.13	0.34
TNM stage	III + IV vs. I + II	< 0.01	3.38	1.83–8.63	< 0.01
Differentiation	well vs. moderate + low	0.08	0.88	0.44–1.52	0.33
Lymph node metastases	positive vs. negative	< 0.01	2.89	1.31–6.84	< 0.01
Distant metastases	positive vs. negative	< 0.01	4.53	2.01–7.94	< 0.01
Serum haptoglobin levels	high vs. low	< 0.01	2.45	1.47–4.49	0.01

Abbreviations: HR = hazards ratio; CI = confidence interval.

## DISCUSSION

In the present study, the diagnostic and prognosis value of serum haptoglobin levels in NSCLC patients were determined and evaluated. We found that serum haptoglobin levels were obviously elevated in NSCLC patients compared with controls. Unfavorable clinicopathological variables, TNM stage, lymph node metastasis, and distant metastasis, were associated with high serum haptoglobin levels. Kaplan-Meier and Cox regression analysis revealed that high haptoglobin levels were correlated with poor overall survival and it could become an independent prognostic factor for NSCLC. Furthermore, ROC analysis displayed that serum haptoglobin level had a potential to distinguish NSCLC patients from normal healthy controls. Considering the haptoglobin test can be easily performed in the conventional clinical biochemistry laboratory by commercially available kit, our results strongly suggest that serum haptoglobin may serve as a useful serum biomarker for the diagnosis and prognosis of NSCLC.

Blood sample is convenient to collection, and the test data can be stable replication during long periods, serological biomarkers have been regarged as valuable indicator in the diagnosis, treatment and prognosis of lung cancer [15]. Accumulating studies in serum sample displayed the pros and cons of usual serum biomarker for NSCLC diagnosis [16–18], while others emphasized their function in disease progression, therapy and prognosis evaluation [19]. *Yang D et al.* measured cytokine contrations by using multiplexed cytokine immunoassays, and found an NSCLC-specific profile of inflammatory mediators, including CXCL10, CXCL11 and CCL20 which would be useful for predicting of therapeutic effects and overall survival [20]. *Wikoff WR et al.* applied a liquid chromatography/mass spectrometry hydrophilic interaction method to analyze a wide rage of serum metabolites, and identified diacetylspermine was a novel serum metabolite with obvious performance in prediagnostic NSCLC [21]. From the clinical investigator's standpoint, CA125, CEA, CYFRA21-1 and SCC still play as the leading serum biomarker in NSCLC and are mainly applied in disease monitoring[17]. Therefore, novel biomarker for diagnosis and prognosis evaluation would assist the doctors to improve the clinical management of NSCLC. The present study was performed to evaluate serum haptoglobin value of diagnostic and prognostic in NSCLC.

In NSCLC, *Hoagland LF et al.* found serum haptoglobin levels significantly elevated in NSCLC patients compared with normal controls, and strongly associated with NSCLC stage [11]. However, this study's subject quantity is not good enough, and no prognostic data. *Park J et al.* utilized stable isotope dilution-multiple reaction monitoring mass spectrometry to investigated the circulating concentration of haptoglobin subunits in 210 NSCLC patients, found serum levels of haptoglobin alpha and beta chains were obviously elevated in NSCLC compared with controls, could be a biomarker for the diagnosis of NSCLC, but lacking of prognostic data [6]. In this study, we detected serum haptoglobin levels in 205 NSCLC patients by commercially available human haptoglobin assay kit, using a fully automated analytical platform, Beckman Coulter AU5800. Our sample's size is adequate, and detect method is very convenient and suitable for the most of primary hospital, which is very important for the vast rural of China and other developing and undeveloped countries.

We verified whether serum haptoglobin levels associated with NSCLC lymph node metastasis or not. The area under the ROCs for the detection of NSCLC lymph node metastasis by serum haptoglobin was 0.609 (sensitivity of 0.672, specificity of 0.554), suggested that haptoglobin might be associated with the progression and poor prognosis of NSCLC. More interesting, another acute-phase protein, C-reactive protein also was found elevated in NSCLC patients, and associated with disease progression and poor prognosis [22, 23]. The precise molecular mechanism for the function of haptoglobin in cancer pathogenesis is still unclear, by now. We speculated that serum haptoglobin levels might be reflected seriousness of infection, which could be caused by tumor necrosis or metastasis. This might suggested that haptoglobin is a potential prognostic biomarker, even the molecular therapeutic target for NSCLC.

However, there were still several limitations in this study. First, this is a single-center study, a large multicenter studies will be needed to adjust the possible biases, to more precisely evaluate the value of serum haptoglobin as a biomarker of NSCLC. Second, the ratio of clinical stage was not ideal. Most of cases were in the advanced stages (III + IV, 70.2%). Third, CA125, CEA, CYFRA21-1 and SCC had not been evaluated; we were not able to compare the diagnostic power of serum haptoglobin with existing biomarker in NSCLC. Last, the exact mechanism of haptoglobin for cancer's tumorigenesis and progression was not clear, by now.

In summary, our results showed that serum haptoglobin levels significantly elevated in NSCLC patients compared with normal healthy controls, and there was a strong association between high serum haptoglobin levels and TNM stages, lymph node metastasis, and distant metastasis. Furthermore, NSCLC patients with higher serum haptoglobin levels had poorer prognosis, suggesting that serum haptoglobin may act as a useful clinical serological biomarkers in diagnosing, progression and prognostic evaluation in NSCLC. These results also suggested that haptoglobin might be a potential therapeutic target for the treatment of NSCLC. However, this hypothesis needed to be explored by further study.

## MATERIALS AND METHODS

### Patient population and specimens

Two hundred and five NSCLC patients of Han Chinese, including one hundred and sixteen patients with adenocarcinoma, seventy-six patients with squamous cell carcinoma, and thirteen patients with other subtypes of NSCLC diagnosed and treated in the First Affiliated Hospital of Sun Yat-sen University in the southern of China, from January 2010 to January 2012 were enrolled in this study. All NSCLC patients' diagnoses were confirmed independently by two pathologists, who reviewed pathological slides of tissue from biopsy or resected specimens. Patients who suffered hyperlipidemia, infection, liver disease, previous malignancy, received antibiotic treatment, or received adjuvant therapy before surgery were excluded from the study. All NSCLC patients received standard treatment with routine therapy, chemotherapy, and radiotherapy after the surgery operation according to NCCN guideline for NSCLC. All patients' histopathological classification was determined according to the WHO criteria, and staged classification was defined according to the 7^th^ edition of UICC TNM staging system [24, 25]. Two hundred and ten normal healthy controls were recruited from healthy unrelated subjects who performed routine yearly health check-up at the Physical Health Examination Centre of our medical center whose age and gender matched subjects who did not have any family history cancer were recruited in this study.

This study was approved by the Human Research Ethics Committee of the First Affiliated Hospital of Sun Yat-sen University according to the guidelines of Helsinki conventions. All participants were informed the information of the purpose of the study and the experimental procedures, and given written informed consent and applied a standardized questionnaire to gather demographic and personal information at the first visit. All subjects with NSCLC were followed up at intervals of one to two months until December 2015, and follow up time ranged from one to sixty months.

### Data collection and laboratory tests

The patients' clinicopathological variables were collected, including age, sex, treatment, histopathological findings, stage, and medication history. Prior to treatment, blood were collected from each subject in the morning between 7 AM and 10 AM for serum tests. To minimize the influence of diet on detecting serum haptoglobin levels, each subjects overnight fast of at least 8 hours before blood collected. Collected blood samples were allowed to clot and serum were separated by centrifugation at 3,000 rpm for 10 min at 4°C, and then determined in the clinical laboratory of the First Affiliated Hospital of Sun Yat-sen University, immediately. Blood fat and hemolytic sample was excluded from the study. Serum haptoglobin levels were determined by a commercially available human haptoglobin assay kit by immunoturbidimetry method (Tridelta PHASE Haptoglobin Assay, County Kildare, Ireland), using a fully automated analytical platform (Beckman Coulter AU5800, Beckman Coulter, Inc., USA). All tests were detected according to the manufacturer's protocols.

### Statistical analyses

All data statistical analyses were performed using Graphpad Prism version 5.0 (GraphPad Software Inc., San Diego, CA, USA) and SPSS version 13.0 (SPSS Inc., Chicago, IL, USA). All variables under normal distribution were shown as the mean ± standard deviation (SD). The differences between groups were determined by Mann-Whitney *U* test and Kruskal-Wallis test. The association between haptoglobin expression levels and various clinicopathological variables in NSCLC were evaluated by using Mann-Whitney *U* test or the Wilcoxon-matched test, and Pearson chi-square test or Fisher's exact test was tested for categorical values. The statistical power was calculated by using the Power and Precision software version 4 (Biostat, NJ, USA). Receiver operating characteristics (ROC) analysis was plotted to determine the sensitivity and specificity of serum haptoglobin levels to discriminate between NSCLC and healthy controls or stratify patients at a high risk of metastasis. The diagnostic power of serum haptoglobin was assessed by sensitivity, specificity, and area under ROC curve (AUC). The cut-off value was determined by the score closest the value under both peak of sensitivity and specificity. Survival rates and curves were determined by the Kaplan-Meier method, and the comparison of differences of survival was evaluated by using the log-rank test. COX regression analysis was used for univariate and multivariate analysis of correlation between clinicopathological variables and overall survival. In all cases, *P* values less than 0.05 were considered statistically significant, and all statistical tests were two-sided.
